# MERS-CoV geography and ecology in the Middle East: analyses of reported camel exposures and a preliminary risk map

**DOI:** 10.1186/s13104-015-1789-1

**Published:** 2015-12-18

**Authors:** Tarian Reeves, Abdallah M. Samy, A. Townsend Peterson

**Affiliations:** Biodiversity Institute, University of Kansas, Lawrence, KS 66045 USA; Faculty of Science, Ain Shams University, Abbassia, Cairo, 11566 Egypt

**Keywords:** MERS-CoV, Virus, Transmission, Camel, Host, Reservoir host, Risk map, Ecological niche

## Abstract

**Background:**

Middle Eastern respiratory syndrome coronavirus (MERS-CoV) has spread rapidly across much of the Middle East, but no quantitative mapping of transmission risk has been developed to date. Moreover, details of the transmission cycle of the virus remain unclear, particularly regarding the role of camels as a reservoir host for human infections.

**Methods:**

We present a first analysis of the environmental circumstances under which MERS-CoV cases have occurred in the Middle East, covering all case occurrences through May 2015, using ecological niche modeling approaches to map transmission risk. We compare the environmental breadth of conditions under which cases have reported camel contacts with that of the broader population of all cases, to assess whether camel-associated cases occur under a more restricted set of environmental circumstances.

**Results:**

We documented geographic and environmental distributions of MERS-CoV cases across the Middle East, and offer preliminary mapping of transmission risk. We confirm the idea that climatic dimensions of camel-associated cases are more constrained and less variable than the broader suite of case occurrences; hence, camel exposure may be a key limiting element in MERS-CoV transmission.

**Conclusion:**

This study offers a first detailed geographic and environmental analysis of MERS-CoV distributions across the Middle East. Results indicated that camel-exposed cases occur under a narrower suite of environmental conditions than non-camel-exposed cases, suggesting perhaps a key role for camels in the transmission of the disease, and perhaps a narrower area of risk for ‘primary,’ camel-derived cases of MERS.

## Background

Middle East respiratory syndrome coronavirus (MERS-CoV) has caused disease in more than 1300 persons across the Middle East [1]. Most cases have been in Saudi Arabia [2], although the virus is known from other countries in the Middle East and North Africa [3–5]; cases exported from the Middle East to Europe and North America [6], and a recent pulse of cases across South Korea, have brought renewed attention to the disease [7]. Clearly, MERS-CoV represents a significant global threat to public health, such that detailed analyses from diverse perspectives are needed.

As the public health community has rushed to understand the etiology and natural history of this disease over the past several years, insights have begun to emerge. A first point is that bats appear to play some sort of ultimate role in the long-term hosting of a diverse community of coronaviruses [e.g., [8]], including virus lineages closely allied to that found in MERS-affected humans [9, 10]. This bat origin and long-term hosting coincides with the apparent conclusions regarding the identity of the long-term host of the preceding SARS-CoV virus that caused disease across East Asia [11].

For MERS-CoV, however, increasing evidence indicates that both camels and humans may play intermediate roles, as both disease victims and reservoirs for further transmission; indeed, no robust evidence indicates direct transmission from bats to humans [see partial and controversial evidence in 9]. Human-to-human transmission has been documented amply [12]; in the Korean situation, for example, transmission appears to be entirely from humans to humans [7]. Contact of various types with camels, such as consumption of raw milk, butchering and cleaning meat, and visiting live animals, has been identified as a significant risk factor, such that camels are seen as a significant reservoir host for MERS-CoV in its transmission to humans (e.g., [13]).

All studies to date of the host ecology and transmission biology of MERS-CoV have been based on case reports, serological studies, and molecular sequence data. The geographic pattern of case occurrences, particularly with respect to the environments manifested at those sites, however, holds potentially rich information about the transmission biology and spatial distribution of transmission risk of diseases such as this one [14]. As such, this paper explores the geographic and environmental circumstances under which MERS-CoV has been transmitted from camels to humans, and where those high-risk circumstances are manifested (i.e., developing a risk map of transmission probabilities). In particular, we test the hypothesis that MERS-CoV cases derived from camel exposure (i.e., as opposed to human-to-human transmission) will have a narrower set of associated environments (i.e., niche breadth), which would be indicative of environmental influences on transmission that can be translated into a map of transmission risk.

## Methods

We conducted an exhaustive search of all mentions of MERS-CoV (search terms “MERS” and “Middle Eastern Respiratory Syndrome”, in ProMED-mail, http://www.promedmail.org/), covering from 2012 through 31 May 2015. The initial number of ProMED posts was 10,248, and each was read carefully by one person (TR), and a form filled out to capture the following information: ProMed archive number, report date, country, state/province, locality, age of person, sex of person, whether person is a healthcare worker, whether the person had other existing co-morbidities, whether the person reported exposure to domestic animals (particularly camels), whether the person reported contact with other MERS cases, and remarks. Each report had to be read carefully to extract information for as many of the fields as possible, and we worked hard not to include duplicate records of cases. Although other, country-specific sources are available regarding MERS (e.g., http://www.moh.gov.sa), we opted for the more consistent, regionwide coverage provided by the global resource.

We next embarked on a long series of steps designed to clean the data, detecting and fixing errors whenever possible, removing any remaining duplicate records, and adding geographic references. These steps involved repeated searches and manipulations of the data set in spreadsheets, as well as use of Microsoft Access to encounter pairs of records with identical information (e.g., for country, city, age, and date) that would signal duplication. These initial cleaning steps reduced the data matrix to 1171 records of MERS-CoV cases.

We then extracted from the data matrix all unique combinations of country, state or province, and locality (93 sites), and assigned latitude-longitude pairs in WGS 1984 geographic coordinates to them via consultation of various online gazetteers (Google Earth, https://www.google.com/earth/; Global Gazetteer version 2.3, http://www.fallingrain.com/world/). In each case, georeferencing was by TR; AMS, a native Arabic speaker, checked all interpretations carefully. We then plotted the geographic coordinates in a GIS (QGIS, version 2.4) to explore further any localities that had suspicious or uncertain georeferences. Of the 93 localities, some did not provide sufficient information or could not be localized with confidence, such that 67 localities and 1113 case reports could be used in our analyses.

We used the WorldClim, version 1.4, ‘bioclimatic’ variable set at 10′ (~17 km) spatial resolution [15] to summarize environments associated with MERS-CoV cases. We used this coarse spatial resolution, and did not take advantage of more information-rich remotely-sensed data resources, in light of the coarse spatial resolution of the input occurrence data—occurrences were generally referenced to city names only, and nothing more specific. We used bioclimatic variables 1–7 and 10–17, omitting variables 8–9 and 18–19, in light of known spatial artifacts in those four variables. Because the remaining 15 variables show considerable intercorrelation and non-independence [16], we used principal components analysis to reduce dimensionality and reduce correlations among variables.

**Fig. 1:**
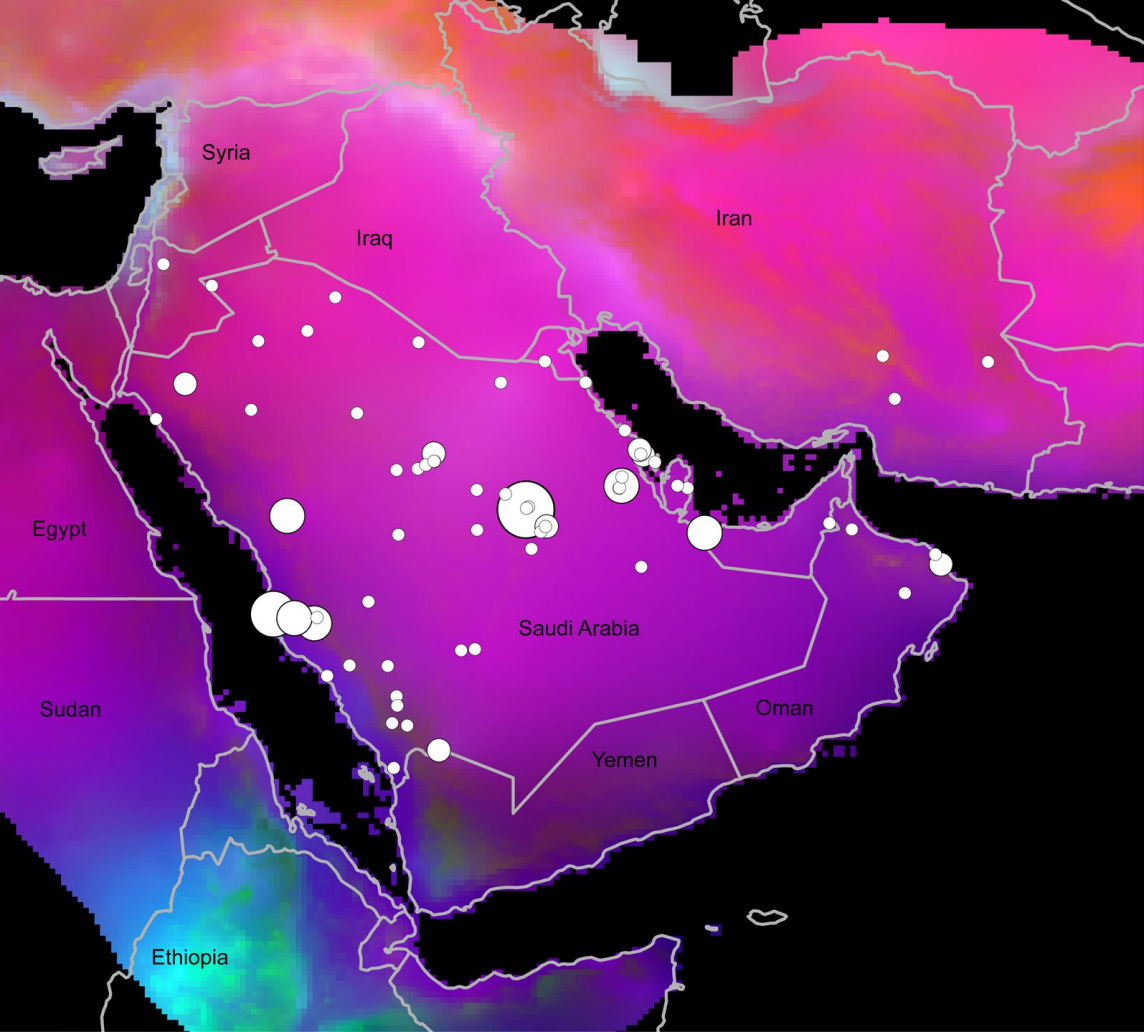
Visualization of environmental variation across the study region, showing sites and numbers of cases. Just for visualization purposes, environments are depicted in a *red*–*green*–*blue* *color* space based on the first three principal components derived from WorldClim bioclimatic variables (i.e., *red* would be maximum value of the first principal component combined with minimum values for the second and third components). *Numbers* of cases are shown in terms of *circle* marker size in a ramp from single cases (*smallest circle*) to 422 (*largest circle*)

Specifically, we first clipped the global rasters to the area within 10° (~1100 km) of known MERS-CoV cases (excluding exported cases in Korea, Europe, and North America), in light of ample evidence that MERS-CoV transmission to humans has concentrated in the Middle East. Next, we ‘stacked’ the 15 bioclimatic raster data layers, and applied a principal components analysis. Because the first five components summarized 99.99 % of the total variance in the original data, we focused on those components only in our analyses. The first three components were plotted in red–green–blue color space for visualization of overall patterns of variation in climate across the region (Fig. 1). To permit exploratory analyses, we used the point sampling tool in QGIS to extract raster data values to each known occurrence point.

To explore patterns of environmental associations, and their implications for the potential geography of MERS-CoV transmission [14], we developed ecological niche models for the disease, creating models for all cases (*N* = 1113), and for only those cases with reported camel contact (*N* = 83). Models were calibrated across the region within 10° of known MERS-CoV cases, as a rough hypothesis of the accessible area for the virus [17]. Using Maxent version 3.3.3k [18], we developed 10 replicate bootstrap analyses for each occurrence data set, and used the median across the 10 as the output (regularization parameter = 1, prevalence = 0.5). We estimated the uncertainty associated with these outputs, pixel by pixel, as the difference between the maximum and minimum values among the 10 random replicate analyses.

To compare niche breadths estimated in the two niche models, we plotted 10,000 random points across the broader study area (of which 7759 fell on land and were included in analyses), and extracted grid values of each of the original bioclimatic variables for each of the points using the point sampling tool in QGIS. We then thresholded each of the niche models using a least training presence thresholding approach, modified to permit an omission rate of *E* = 0.1 [19], such that we identified the highest raw suitability level that included 90 % of the data with which the model was calibrated in the suitable area. We then discarded all random points not identified as suitable in either of the models, leaving 5936 points for analysis. Finally, as fewer points were identified as suitable in the model based on camel exposures (4811 points), we rarefied the larger set of points identified as suitable in the model based on all MERS-CoV cases (5707 points) down to the number of points suitable in the camel model, and calculated the standard deviation of each environmental variable associated with points in each data set. Based on direct counts of the observed standard deviation from the camel-based model with those among the 100 random replicates drawn from the unrestricted model, we tested whether the niche estimated based on camel exposures was narrower than that based on all cases.

## Results

**Fig. 2:**
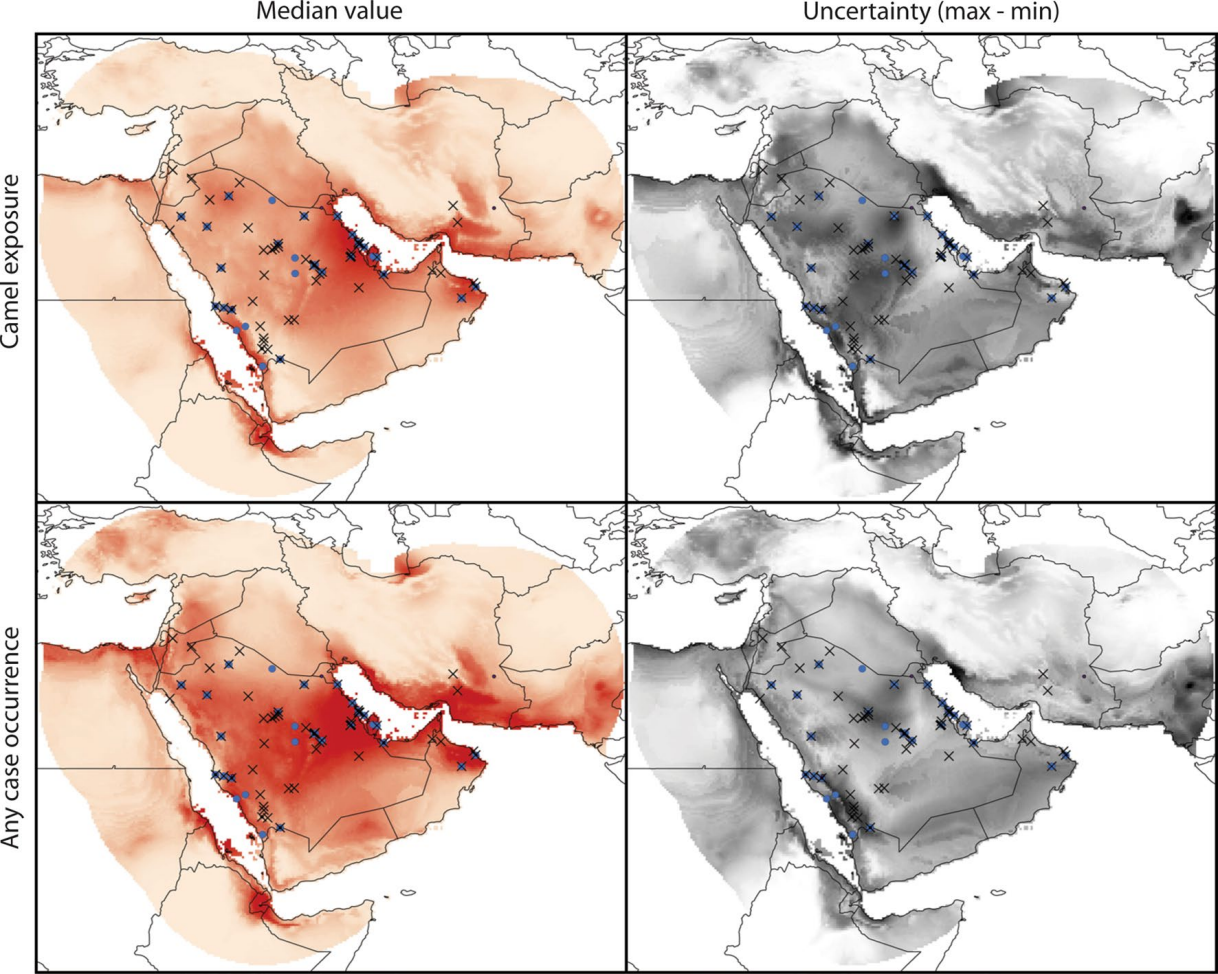
Visualization of ecological niche model outputs in terms of median prediction of suitability for MERS-CoV transmission to humans (*left column*; *light orange* low suitability, *dark orange* high suitability), and the uncertainty associated with those predictions estimated as the range of suitability values (pixel by pixel) among the 10 bootstrapped random replicate models (*right column*; *white* low uncertainty, *black* high uncertainty). *Dots* represent camel-exposed cases; X’s represent any case occurrence

Our MERS-CoV case-occurrence data set reflected a clear concentration of cases in Saudi Arabia (that is, among Middle Eastern cases only; Fig. 1), although a few cases have been detected in neighboring countries (United Arab Emirates, Qatar, Oman, Jordan, Iran). This concentration was reflected in the ecological niche model outputs (Fig. 2): the model based on reported camel exposure showed highest suitability in the northeastern and southwestern parts of the Arabian Peninsula, as well as adjacent areas of southwestern Asia and northeastern Africa. The model based on all occurrences, on the other hand, identified a broader suitable area across the central portion of the Arabian Peninsula and adjacent areas of Asia and Africa. High uncertainty in these predictions, in both cases, coincided with areas of high modeled suitability (Fig. 2), such that confidence in model predictions is not uniformly high.

**Fig. 3:**
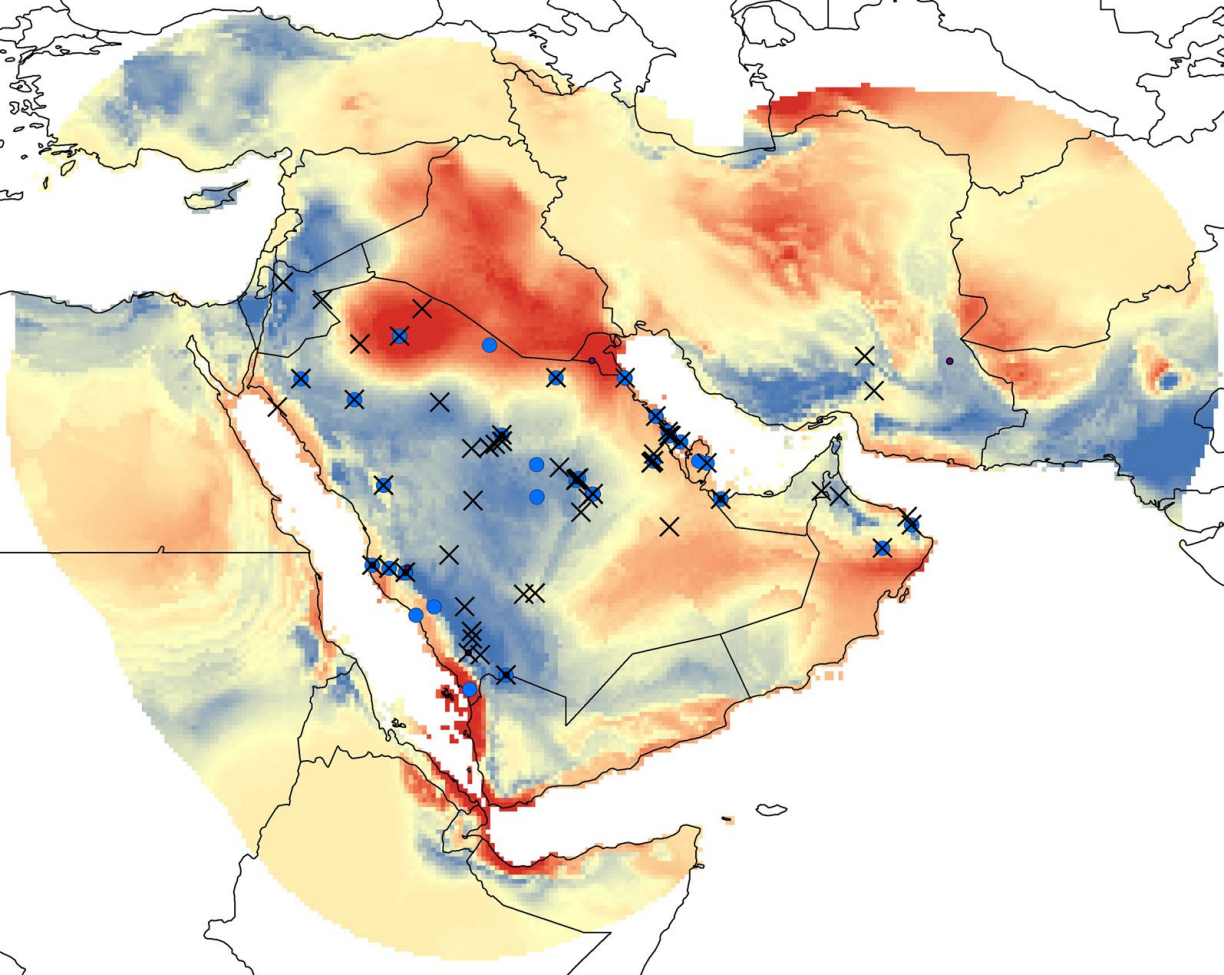
Map of difference between the two ecological niche models (one based on reported camel exposure versus one based on all reported cases). *Red* areas emphasized in the camel-exposed analysis, *blue* areas emphasized in the any-case analysis. *Dots* represent camel-exposed cases; X’s represent any case occurrence

The outputs of the two ecological niche models contrasted in their spatial predictions across the study region. Figure 3 shows the difference between the two outputs: the model based on camel exposures emphasized the northern and southern coastal parts of the Arabian Peninsula, and de-emphasized the central and western parts. Clearly, the two models emphasize different regions, which reflects different environmental regimes underlying the occurrence data that drive the model outputs.

**Fig. 4:**
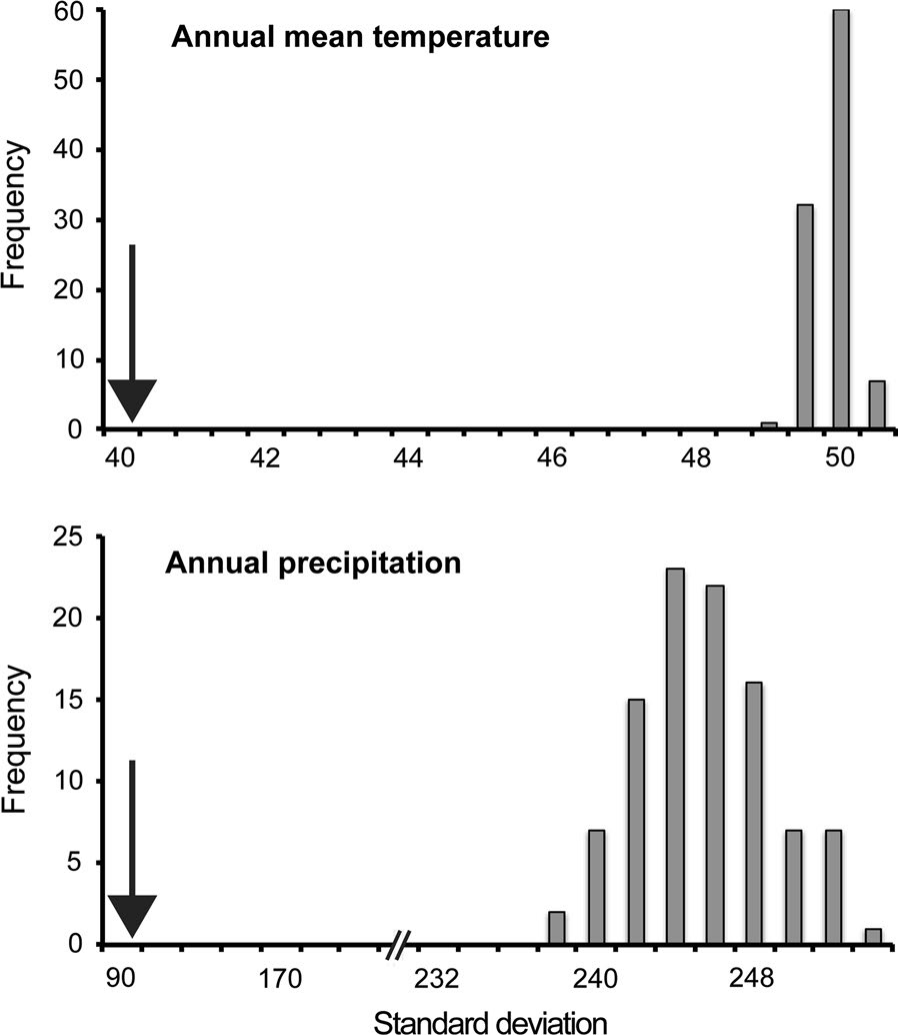
Standard deviations (niche breadths) of values of annual mean temperature (*top*; in °C × 10) and annual precipitation (*bottom*; in mm) across random points identified as suitable for MERS-CoV transmission to humans. The *black arrow* indicates the standard deviation of the model based on camel exposures, and the frequency histograms show the rarefied resampling of the model based on all occurrences. Note the broken scale in the *bottom panel*: intervals are 20 units in the *left portion*, and 2 units in the *right portion*

To explore relative niche breadths of models based on reported camel exposure versus the broader suite of possible exposure regimes that could produce MERS-CoV cases, we developed detailed comparisons of ecological niche breadths. That is, we applied 10 % omission thresholds of 0.05357 for models based on camel exposures, and 0.02958 for models based on all occurrences (note that these thresholds are driven by the relationship between calibration data and model outputs) to convert the two to binary models. We then related these two predictions to annual mean temperature and annual precipitation via the random points across the study region. The rarefied points for annual mean temperature in the camel-based model had a standard deviation of 40.5, whereas those for the model based on any occurrence ranged 49.0–50.2; for annual precipitation, the camel-based model had a standard deviation of 94.7, whereas those for the any-occurrence model ranged 236.2–253.1 (Fig. 4). Hence, with respect to both temperature and precipitation, the diversity of environments indicated as suitable based on camel-based exposures was significantly less than the diversity of environments so identified based on all occurrences (*P* < 0.01, in both cases).

## Discussion

MERS-CoV has had a dramatic, if brief, history, in which it has emerged in the Middle East, and spread to at least four continents in just 3 years. Although many analyses have been published regarding various aspects of the distribution [3, 5], epidemiology [20], evolution [21], and phylogeny [22], major and significant knowledge gaps exist regarding this disease [23]. This contribution offers a novel approach: we tested and corroborated the hypothesis that transmission from camels to humans occurs under a quite-restricted set of environmental circumstances. We also produce a first view of regionwide suitability for MERS-CoV transmission to humans, focusing in the Middle East, as that is the region in which the disease has the longest history.

Our ecological niche models represent and reconstruct the relationship between the occurrence data and the environmental data; although we are intrigued by the spatial and environmental patterns that we detected, we are nonetheless cognizant of several limitations of this approach. First of all, the occurrence data are quite crude in their geographic localization: because MERS-CoV exposure and infection are rather coarse and diffuse, the occurrence data cannot be georeferenced more finely than multiple kilometers, which limits the spatial detail in our predictions, and constrains us from using remote-sensing data to achieve improved spatial resolution [24].

Second, reporting of exposure patterns in MERS-CoV cases is rather uneven and unreliable. That is, fewer than 10 % of the overall suite of cases reported contact with camels; this small number either reflects a dominant role of human-to-human transmission in the etiology of this disease, or lack of recognition and reporting of camel contacts among other cases. On the other hand, some portion of the camel contacts reported may represent ‘red herrings’: the contact reported may not have been the source of the infection.

Given these shortcomings in the data, we were impressed by the clarity of the results that we obtained. Our analyses pointed clearly to a restricted set of environmental conditions under which MERS-CoV is transmitted from camels to humans: niche breadth in the camel-exposed models was strikingly restricted relative to that manifested in the any-occurrence models. This better definition of a ‘transmission niche’ may reflect a narrower suite of environmental conditions under which camel-to-human MERS-CoV transmission can occur.

This well-defined ecological niche can in turn be used to explore the potential geography of the transmission phenomenon [14]. We documented an intriguing contrast between the overall transmission geography and that associated with camel exposure. The implication, then, is that circumscribed portions of the Arabian Peninsula can be identified in which camel-to-human MERS-CoV transmission may be particularly likely.

The results of this study also point to a number of research and data gaps in the knowledge of the disease. That is, we note considerable variation and incompleteness of reporting of cases: much more could be learned about this disease (and others) were data reporting to be made more standard and uniform in quality. Detailed geographic sampling of bat, camel, and human populations across the study region would enrich the environmental picture considerably, in addition to permitting detection of reporting filters and other biases affecting the picture of the disease’s geography and ecology [25]; we also see considerable need for sampling and testing more broadly across North Africa and Sub-Saharan Africa [26]. These improvements would offer greatly improved insights into the questions on which this analysis centers.

## Availability of supporting data

The data set supporting the results of this article is available in the LabArchives repository, DOI: 10.6070/H4DR2SHR.
